# Supplementary material for: Assessing least-cost mitigation methods for environmental phosphorus loading of different pasture-based and housed dairy production systems in Great Britain
Source: PLoS One. 2025 Mar 26;20(3):e0319919. doi: 10.1371/journal.pone.0319919 (PMC11940655; doi:10.1371/journal.pone.0319919)
Supplement: S1 Table — aGenerated using average (mean for continuous and mode for categorical) data of 20 surveyed farms, bTotal cost is the sum of capital and operational costs., cTotal cost and reduction in environmental P loading may vary when evaluating mitigation methods individually compared to together (DOCX) [file pone.0319919.s001.docx]

| **Mitigation method** | **P loss reduction (%)** | **Total Cost^b^ (£)** |
| --- | --- | --- |
| **Use correctly-inflated low ground pressure tyres on machinery** | 1.3 | -2373 |
| **Leave out winter stubbles** | 0.7 | 344 |
| **Unfertilised cereal headlands** | 0.0 | 380 |
| **Management of arable field corners** | 1.3 | 383 |
| **Management of in-field ponds** | 0.5 | 35 |
| **Establish new hedges** | 0.0 | 279 |
| **Do not spread FYM at hgih risk times** | 0.8 | 16 |
| **Do not spread slurry or poultry manure at high-risk times** | 4.0 | 16 |
| **Do not apply manure to high-risk area** | 0.0 | 0.0 |
| **Cover solid manure stores with sheeting** | 0.3 | 171 |
| **Store solid manure heaps on an impermeable base and collect effluent** | 1.4 | 1348 |
| **Extend the grazing season** | -7.0 | -9506 |
| **Do not apply P fertiliser to high index soils** | 1.2 | -730 |
| **Use manafactured fertiliser placement technology** | 0.0 | -143 |
| **Integrate fertilise and manure nutrient supply** | 0.0 | -13928 |
| **Use a fertiliser recommendation systems** | 0.0 | -427 |
| **Make use of improved genetics in livestock** | 0.6 | -25586 |
| **Loosen compacted soils in grassland fields** | 12.5 | 2417 |
| **Establish in-feild grass buffer strips** | 3.5 | 176 |
| **Manage over winter tramlines** | 0.1 | 7 |
| **Leave autumn seedbeds rough** | 0.0 | 151 |
| **Cultivate and drill across the slope** | 0.2 | 58 |
| **Unfertilised cereal headlands** | 0 | 380 |
| **Cultivate compacted tillage soils** | 3.7 | 421 |
| **Construct troughs with concrete base** | 3.6 | 726 |
| **Farm track management** | 0 | 46 |
| **Total^c^** | **28.7** | **- 45339** |

Supporting 1 Table
