# Supplementary material for: Assessing least-cost mitigation methods for environmental phosphorus loading of different pasture-based and housed dairy production systems in Great Britain
Source: PLoS One. 2025 Mar 26;20(3):e0319919. doi: 10.1371/journal.pone.0319919 (PMC11940655; doi:10.1371/journal.pone.0319919)
Supplement: S2 Table — aGenerated using average (mean for continuous and mode for categorical) data of 7 surveyed farms, bTotal cost is the sum of capital and operational costs, cTotal cost and reduction in environmental P loading may vary when evaluating mitigation methods individually compared to together (DOCX) [file pone.0319919.s002.docx]

| **Mitigation method** | **P loss reduction (%)** | **Total Cost^b^ (£)** |
| --- | --- | --- |
| **Increase use of maize silage** | - 0.3 | -1665 |
| **Use correctly-inflated low ground pressure tyres on machinery** | 3.2 | -2438 |
| **Management of arable field corners** | 3.1 | 644 |
| **Management of in-field ponds** | 1.4 | 53 |
| **Do not spread slurry or poultry manure at high-risk times** | 0.3 | 62 |
| **Construct water troughs with concrete base** | 2.3 | 451 |
| **Extend the grazing season** | -6.2 | -9613 |
| **Do not apply P fertiliser to high index soils** | 2.6 | -630 |
| **Integrate fertiliser and manure nutrient supply** | 0.0 | -34329 |
| **Use a fertiliser recommendation systems** | 0.2 | -1548 |
| **Make use of improved genetics in livestock** | 0.5 | -26052 |
| **Establish riparian buffer strips** | 3.8 | 183 |
| **Leave autumn seedbeds rough** | 0.2 | 522 |
| **Establish in-feild grass buffer strips** | 8.0 | 271 |
| **Total^c^** | **19.1** | **-74089** |

Supporting 2 Table
