# Supplementary material for: Assessing least-cost mitigation methods for environmental phosphorus loading of different pasture-based and housed dairy production systems in Great Britain
Source: PLoS One. 2025 Mar 26;20(3):e0319919. doi: 10.1371/journal.pone.0319919 (PMC11940655; doi:10.1371/journal.pone.0319919)
Supplement: Attachment — Submitted filename: Reviewer comments (Submission PONE-D-24-27234).pdf [file pone.0319919.s005.pdf]

Journal: PLOS One

Manuscript title: Assessing least cost mitigation methods for environmental phosphorus loading of different pasture-based and housed dairy production systems in Great Britain.

Manuscript No.: PONE-D-24-27234

Reviewer comments to authors

This study investigated environmental P loading (EPL) from dairy farms operating on pasture based and housed systems using the FARMSCOPER tool. The results indicated that house systems generally had a higher EPL than pasture-based systems and positively correlated with milk production. The model also showed that mitigation measures can significantly reduce EPL's and are cost effective.

Overall assessment

This study is interesting and emphasises the need to update the FARMSCOPER tool with more data sets. The report is well written, easy to follow, and I have only a very few suggested minor comments for the authors:-

- Abstract - it might be worth noting here that there is a need to include more specific farm data to improve the FARMSCOPER dataset (as noted throughout the report).
- L280 – 'range = 13.8 to 583.6…' - a comment to clarify the wide range would be useful here i.e. which farms were low, and which were high etc.
- L282 - a comment to outline what these mitigation methods are would be helpful here (e.g. a reference to Table 3 perhaps).
- L286 – 'maximum scenario' – this term has been used throughout the report, but I am unclear as to what it means.
- L315 – include all R^2 values to 1 decimal place for consistency.
- L366 – 'potential annual savings…' – a comment here as to how such savings can be achieved would be useful.
- L430 – 'a' or 'as'?

Overall, very interesting study – well done!
